# CT-Visible Venous Fat Embolus Associated With a Femoral Shaft Fracture in a Polytrauma Patient: A Case Report

**DOI:** 10.7759/cureus.109475

**Published:** 2026-05-23

**Authors:** El Mahdi Choukri, Ali Meqor, Jalal El Kidari, Asmae Oulad Amar, Siham Alaoui Rachidi

**Affiliations:** 1 Diagnostic and Interventional Radiology, Mohammed VI University Hospital, Tangier, MAR; 2 Radiology, Faculty of Medicine and Pharmacy, Abdelmalek Essaâdi University, Tangier, MAR

**Keywords:** computed tomography, fat embolism syndrome, femoral shaft fracture, iliofemoral vein, polytrauma, splenic injury, venous fat embolus, whole-body trauma ct

## Abstract

Venous fat emboli are uncommon but important radiological findings in patients with long-bone fractures, and direct visualization on computed tomography (CT) is rarely reported. We report the case of a 34-year-old man with polytrauma, reported to be hemodynamically stable, admitted after a motorcycle road traffic accident. Initial radiography demonstrated a complete, displaced fracture of the proximal third of the left femoral shaft. Whole-body trauma CT revealed a fat-attenuation intravascular lesion within the distal left external iliac vein extending into the proximal left common femoral vein, with an attenuation value of approximately -81 Hounsfield units, consistent with a venous fat embolus. The lesion persisted in the same venous location across the available non-contrast, arterial-phase, and portal venous-phase images. Thoracic CT angiography showed no pulmonary arterial filling defect or acute pulmonary parenchymal abnormality on the available images. Abdominopelvic CT also demonstrated a superior polar splenic laceration measuring more than 3 cm in depth, associated with perisplenic hemoperitoneum, consistent with grade III splenic injury according to the American Association for the Surgery of Trauma classification. No definite active contrast extravasation was identified on the available arterial, portal venous, and delayed-phase images. Orthopedic fixation of the femoral fracture was subsequently performed; however, detailed follow-up data, including respiratory evolution, neurological status, laboratory data, splenic injury management, and discharge outcome, were not documented in the available medical record. This case highlights the importance of systematic venous assessment on whole-body trauma CT in patients with long-bone fractures and emphasizes the distinction between a CT-visible venous fat embolus and fat embolism syndrome, which remains a clinical diagnosis requiring appropriate clinical correlation.

## Introduction

Fat embolization is a recognized complication of long-bone fractures, particularly femoral shaft fractures, and may have clinical relevance because an embolic material can migrate through the venous circulation toward the pulmonary vascular bed. However, direct visualization of a macroscopic venous fat embolus on computed tomography (CT) is uncommon and has been only rarely reported [1-3]. On CT, a venous fat embolus appears as an intravascular filling defect with fat attenuation, typically showing negative Hounsfield unit values, reflecting its fatty composition. Recognition of this finding is important because embolic migration may theoretically lead to pulmonary fat embolism and, in the appropriate clinical setting, may be associated with fat embolism syndrome. However, the presence of a CT-visible venous fat embolus alone does not establish the diagnosis of fat embolism syndrome, which remains a clinical diagnosis requiring appropriate respiratory, neurological, and systemic correlation [4].

Whole-body trauma CT plays a central role in the evaluation of polytrauma patients by allowing simultaneous assessment of skeletal, thoracic, abdominal, pelvic, and vascular injuries. In patients with long-bone fractures, a systematic review of the venous system is important because rare fat-attenuation intravascular emboli may otherwise be overlooked, particularly when attention is focused on fractures, solid-organ injuries, or arterial bleeding. Associated abdominal injuries, including splenic trauma, should also be characterized when present because injury severity, hemodynamic status, and the presence or absence of active bleeding influence monitoring and management decisions [5,6].

We report the case of a 34-year-old man with polytrauma, reported as clinically stable, with a displaced left femoral shaft fracture, a CT-visible venous fat embolus in the left iliofemoral venous axis, and an associated grade III splenic injury. This case highlights the value of systematic venous assessment on whole-body trauma CT and emphasizes the distinction between radiological detection of a venous fat embolus and the clinical diagnosis of fat embolism syndrome.

## Case presentation

A 34-year-old man was admitted to the adult emergency department following a motorcycle road traffic accident. No loss of consciousness or vomiting was reported. On initial clinical assessment, the patient was reported as hemodynamically stable and presented with painful inspiration, complete functional impairment of the left lower limb associated with pain, and pain during mobilization of the left shoulder. Objective admission vital signs, including blood pressure, heart rate, respiratory rate, oxygen saturation, temperature, and Glasgow Coma Scale score, were not documented in the available medical record. Dyspnea, documented hypoxemia, respiratory deterioration, focal neurological symptoms, petechial rash, and laboratory abnormalities suggestive of fat embolism syndrome were also not documented in the available medical record.

Initial radiography of the left femur demonstrated a complete, displaced fracture of the proximal third of the left femoral shaft, with fragment overlap and small adjacent osseous fragments (Figure 1).

**Figure 1:**
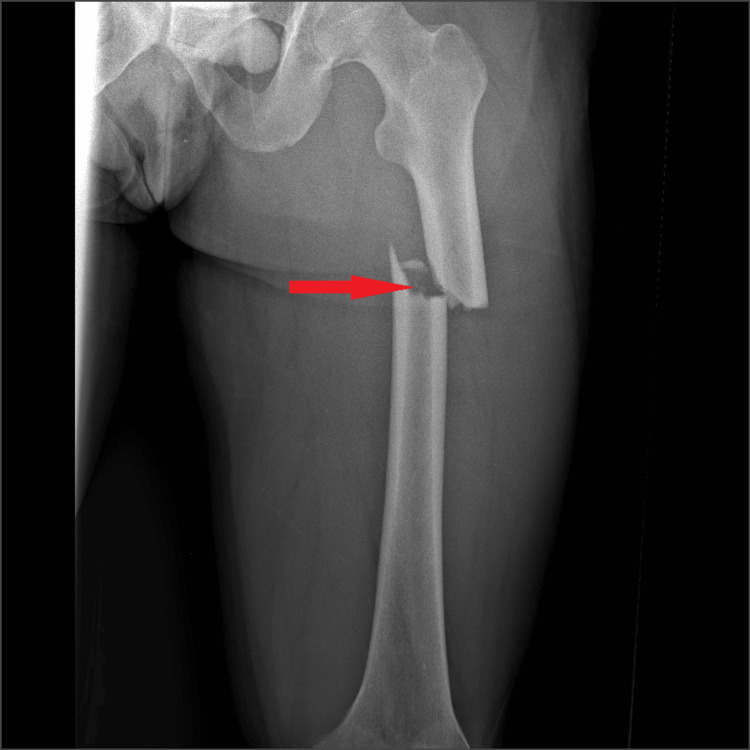
Initial frontal radiograph of the left femur showing a displaced proximal femoral shaft fracture. Frontal radiograph demonstrates a complete displaced fracture of the proximal third of the left femoral shaft, with fragment overlap and small adjacent osseous fragments. The red arrow indicates the fracture site.

A whole-body trauma CT scan, including head, cervical spine, thoracic, abdominal, and pelvic acquisitions before and after intravenous contrast administration, was performed. The available CT acquisition phases included non-contrast, arterial-phase, portal venous-phase, and delayed-phase imaging. Contrast volume, exact acquisition timing, and slice thickness were not documented in the available imaging record. The examination was technically limited by patient-positioning artifacts related to the patient’s arm visible within the field of view, particularly on upper abdominal images. Head CT showed no acute post-traumatic intracranial hemorrhage, midline shift, or cerebral herniation. Cervical spine CT showed preserved craniocervical alignment, preserved C1-C2 relationship, normal posterior vertebral alignment, and no traumatic vertebral body height loss.

Thoracic CT angiography showed no pneumothorax, hemothorax, pneumomediastinum, mediastinal hematoma, pericardial effusion, pulmonary arterial filling defect, or post-traumatic pulmonary parenchymal abnormality on the available images. Within the limits of the technically limited examination, no CT evidence of pulmonary embolism or acute pulmonary fat embolism was identified at the time of imaging.

Abdominopelvic CT demonstrated moderate hemoperitoneum, located mainly in the perisplenic and perihepatic spaces, both paracolic gutters, and the pelvis. The spleen was normal in size but showed a large superior polar laceration measuring more than 3 cm in depth, associated with additional small peripheral lacerations. The specific American Association for the Surgery of Trauma criterion supporting grade III splenic injury was the laceration depth greater than 3 cm [5]. No definite active contrast extravasation was identified on the available arterial, portal venous, and delayed-phase images. Mild hemorrhagic infiltration of the left renal fossa and ipsilateral anterior and posterior pararenal fascia was also noted. No pneumoperitoneum was detected, and the liver, pancreas, kidneys, and adrenal glands showed no traumatic abnormality.

A fat-density intravascular lesion was identified within the distal left external iliac vein, extending into the proximal left common femoral vein, consistent with a venous fat embolus (Figure 2). The exact lesion length was not documented in the available imaging report. Its fatty composition was supported by an attenuation value of approximately -81 Hounsfield units, measured within the fat-density intravascular lesion on the magnified non-contrast axial CT image. The attenuation measurement was obtained using an elliptical region of interest placed within the fat-density intravascular lesion on the magnified non-contrast axial CT image, with an area of approximately 0.11 cm², height of 3.56 mm, and width of 3.93 mm. The lesion persisted in the same left iliofemoral venous location across the available non-contrast, arterial-phase, and portal venous-phase images, with no documented change in position between phases.

**Figure 2:**
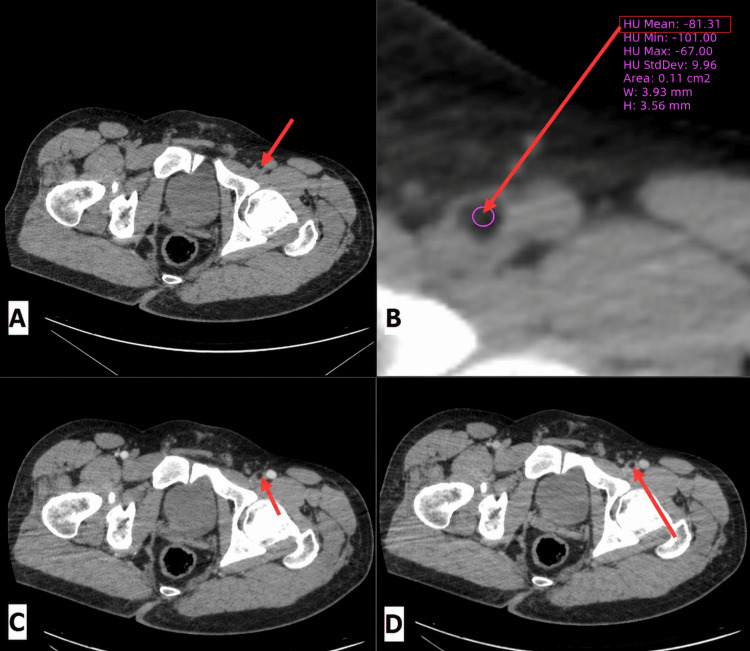
Axial CT characterization of a persistent venous fat embolus within the left iliofemoral venous axis. (A) Non-contrast axial CT image shows a fat-density intravascular focus within the distal left external iliac vein/proximal left common femoral vein (red arrow). (B) Magnified non-contrast axial CT image shows an attenuation measurement of approximately -81 Hounsfield units within the fat-density intravascular lesion, supporting its fatty composition. The measurement was obtained using an elliptical region of interest placed within the lesion, with an area of approximately 0.11 cm², height of 3.56 mm, and width of 3.93 mm. (C) Contrast-enhanced arterial-phase axial CT image demonstrates persistence of the same fat-density intravascular lesion in the same venous location (red arrow), with no associated arterial abnormality identified. (D) Portal venous-phase axial CT image confirms persistence of the fat-density intravascular lesion in the same left iliofemoral venous location (red arrow). CT: computed tomography; HU: Hounsfield units

Multiplanar reconstructions confirmed the intravascular location and longitudinal extension of the venous fat embolus along the left iliofemoral venous axis (Figure 3).

**Figure 3:**
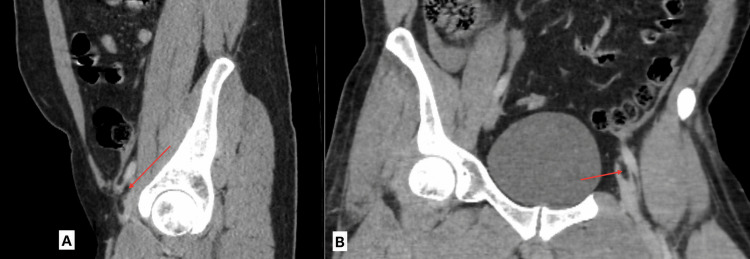
Multiplanar CT confirmation of a persistent venous fat embolus along the left iliofemoral venous axis. (A) Sagittal portal venous-phase CT reconstruction demonstrates the fat-density intravascular lesion within the left iliofemoral venous axis (red arrow). (B) Coronal portal venous-phase CT reconstruction confirms its intravascular location and longitudinal extension along the distal left external iliac vein and proximal left common femoral vein (red arrow). The exact lesion length was not documented in the available imaging report. CT: computed tomography

The associated splenic lesions were illustrated on non-contrast, arterial-phase, and portal venous-phase CT images, confirming a superior polar splenic laceration with adjacent perisplenic hemoperitoneum (Figure 4). Delayed-phase images, not shown, confirmed the absence of definite active contrast extravasation.

**Figure 4:**
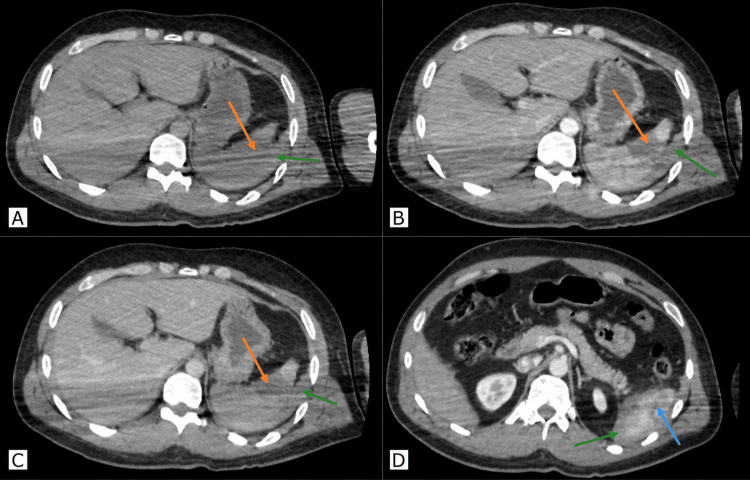
CT appearance of the associated grade III splenic injury without definite active contrast extravasation. (A) Non-contrast axial CT image shows traumatic splenic parenchymal abnormality corresponding to the superior polar splenic injury (orange arrow), with adjacent perisplenic hemoperitoneum (green arrow). (B) Contrast-enhanced arterial-phase CT image demonstrates the splenic injury/laceration (orange arrow) and associated perisplenic hemoperitoneum (green arrow). (C) Portal venous-phase CT image confirms the splenic laceration and surrounding perisplenic hemoperitoneum. (D) Arterial-phase CT image better depicts the superior polar splenic laceration (blue arrow), associated with perisplenic hemoperitoneum (green arrow). No definite active contrast extravasation is identified on the illustrated arterial and portal venous-phase images. Delayed-phase images, not shown, also confirmed the absence of definite active contrast extravasation. CT: computed tomography

Additional findings included hemorrhagic infiltration of the subcutaneous soft tissues in the lumbar region and along the left lateral abdominal wall.

Orthopedic fixation of the left femoral fracture was subsequently performed; however, the exact timing of fixation relative to CT, the type of fixation, and the perioperative course were not documented in the available medical record. The management strategy for the splenic injury, including whether transfusion, angiography, surgery, or conservative management was required, was also not documented. Respiratory evolution, oxygen saturation data, neurological follow-up, laboratory follow-up, follow-up imaging, and discharge outcome were not documented in the available medical record. Therefore, the case was interpreted as a radiological diagnosis of CT-visible venous fat embolus associated with a femoral shaft fracture, without available clinical evidence to establish fat embolism syndrome.

## Discussion

Venous fat emboli are uncommon radiological findings in trauma imaging, although fat embolization is a recognized consequence of long-bone fractures, particularly femoral shaft fractures. In most cases, fat emboli are not directly visualized on imaging, and the diagnosis is considered clinically when patients develop respiratory, neurological, or systemic manifestations. In contrast, macroscopic venous fat emboli may occasionally be detected on CT as fat-attenuation intravascular filling defects, particularly near pelvic or lower-limb fractures. The available literature remains limited and consists mainly of isolated CT-detected venous cases, including reports in the common femoral vein, lower-extremity veins, popliteal vein, and common iliac vein after trauma [1-3,7-10]. This rarity supports the educational value of systematic venous review during trauma CT in patients with long-bone fractures.

The present case illustrates this rare CT finding in a polytrauma patient with a displaced femoral shaft fracture. The intravascular lesion was identified within the distal left external iliac vein, extending into the proximal left common femoral vein. Its attenuation value of approximately -81 Hounsfield units, measured within the fat-density intravascular lesion on the magnified non-contrast axial CT image, supported its fatty composition rather than a soft-tissue thrombus. The attenuation measurement was obtained using an elliptical region of interest placed within the fat-density intravascular lesion, with an area of approximately 0.11 cm², height of 3.56 mm, and width of 3.93 mm. The exact lesion length was not documented in the available imaging report. The lesion persisted in the same left iliofemoral venous location across the available non-contrast, arterial-phase, and portal venous-phase images, with no documented change in position between phases. Multiplanar reconstructions were useful to confirm the intravascular location and longitudinal extension of the embolus along the left iliofemoral venous axis. This point is important because small fat-attenuation intravascular foci may otherwise be overlooked or misinterpreted, particularly in technically limited trauma CT examinations.

A key distinction must be made between a CT-visible venous fat embolus and fat embolism syndrome. Fat embolism syndrome is a clinical diagnosis that typically requires respiratory impairment, neurological manifestations, petechial rash, or supportive laboratory and imaging findings [4,11]. In the present case, pulmonary fat embolism was considered because of the venous intravascular fat-density lesion and the associated femoral shaft fracture. Thoracic CT angiography showed no pulmonary arterial filling defect and no acute pulmonary parenchymal abnormality on the available images. However, the examination was technically limited by patient-positioning artifacts related to the patient’s arm visible within the field of view, and respiratory evolution, oxygen saturation data, neurological follow-up, petechial rash assessment, laboratory follow-up, and clinical outcome were not documented in the available medical record. Therefore, the finding should be reported as a CT-visible venous fat embolus associated with femoral shaft fracture, without establishing a diagnosis of fat embolism syndrome or excluding later clinical evolution.

Detection of a venous fat embolus on trauma CT has practical implications for radiological reporting and multidisciplinary trauma care. Because embolic migration may theoretically lead to pulmonary fat embolism and, in the appropriate clinical context, fat embolism syndrome, this finding should prompt careful evaluation of the pulmonary arteries and lung parenchyma, correlation with respiratory and neurological status, and timely communication with the trauma team. Reported management approaches in previous cases have varied and may include clinical observation, orthopedic stabilization, anticoagulation, or inferior vena cava filter placement depending on embolus location, mobility or migration, associated injuries, and overall clinical status [1-3,7-10]. However, there is no standardized management strategy specifically for incidentally detected CT-visible venous fat emboli. Therefore, recognition of this finding should mainly support individualized multidisciplinary assessment and clinical vigilance rather than imply a uniform treatment pathway.

This case was also associated with splenic trauma. The splenic injury consisted of a superior polar laceration measuring more than 3 cm in depth, associated with perisplenic hemoperitoneum. The specific American Association for the Surgery of Trauma criterion supporting grade III splenic injury was laceration depth greater than 3 cm [5]. No definite active contrast extravasation was identified on the available arterial, portal venous, and delayed-phase images. Accurate grading remains important because splenic injury severity, hemodynamic status, hemoperitoneum extent, and the presence or absence of active bleeding are key factors influencing monitoring and management decisions [5,6]. However, the management strategy for the splenic injury in this case, including whether transfusion, angiography, surgery, or conservative management was required, was not documented in the available medical record.

This case has several limitations. Objective admission vital signs, Glasgow Coma Scale score, oxygen saturation data, detailed respiratory evolution, neurological follow-up, petechial rash assessment, laboratory follow-up, exact timing and type of femoral fixation, perioperative course, splenic injury management, follow-up imaging, and discharge outcome were not documented in the available medical record. In addition, the available imaging record did not document the exact CT contrast volume, acquisition timing, slice thickness, or exact lesion length. Therefore, clinical progression toward fat embolism syndrome could not be fully assessed, and later pulmonary fat embolism or fat embolism syndrome cannot be excluded clinically. Despite these limitations, the case remains educational because it demonstrates a rare CT-visible venous fat embolus associated with femoral shaft fracture and highlights the importance of systematic venous assessment on whole-body trauma CT in patients with long-bone fractures.

## Conclusions

This case highlights the importance of systematic venous assessment on whole-body trauma CT in patients with long-bone fractures. A fat-attenuation intravascular lesion with negative Hounsfield unit values within the iliofemoral venous axis should suggest a venous fat embolus, particularly in the setting of femoral shaft fracture. This radiological finding should be clearly distinguished from fat embolism syndrome, which remains a clinical diagnosis requiring respiratory, neurological, and systemic correlation and cannot be established on CT appearance alone.

The associated grade III splenic injury further emphasizes the value of whole-body trauma CT in detecting, characterizing, and grading concomitant traumatic injuries that may influence clinical monitoring and management. Because detailed clinical follow-up, respiratory evolution, neurological assessment, laboratory follow-up, splenic injury management, and discharge outcome were not documented in the available medical record, the clinical significance of the venous fat embolus in this patient could not be fully assessed. Therefore, early recognition and clear reporting of CT-visible venous fat emboli may support multidisciplinary communication and appropriate clinical vigilance in polytrauma patients, rather than imply a proven effect on clinical outcomes.
